# Polyphenol Analysis and Antibacterial Potentials of Twig Extracts of *Salix aurita*, *S. pyrolifolia*, and *S. caprea* Growing Naturally in Finland

**DOI:** 10.3390/ijms252211978

**Published:** 2024-11-07

**Authors:** Eunice Ego Mgbeahuruike, Enass Salih, Stella Prévost-Monteiro, Nina Sipari, Henry Väre, Riitta Julkunen-Tiitto, Pia Fyhrqvist

**Affiliations:** 1 Division of Pharmaceutical Biosciences, Faculty of Pharmacy, University of Helsinki, 00100 Helsinki, Finland; enass.salih@helsinki.fi (E.S.); pia.fyhrquist@helsinki.fi (P.F.); 2 Faculty of Pharmaceutical Sciences, University of Bordeaux, 33076 Bordeaux, France; sprevostmonteiro@gmail.com; 3 Viikki Metabolomics Unit, Organismal and Evolutionary Biology Research Programme, Faculty of Biological and Environmental Sciences, University of Helsinki, 00100 Helsinki, Finland; nina.sipari@helsinki.fi; 4 Botanical Museum, Finnish Museum of Natural History, University of Helsinki, 00100 Helsinki, Finland; henry.vare@helsinki.fi; 5 Faculty of Science and Forestry, Department of Environmental and Biological Sciences, University of Eastern Finland, 80100 Joensuu, Finland; riitta.julkunen-tiitto@uef.fi

**Keywords:** antibacterial activity, polyphenols, *Salix aurita*, twigs, UPLC-PDA-QTOF/MS, procyanidins, *Salix caprea*, *Salix pyrolifolia*

## Abstract

*Salix* species have been used in traditional medicine to treat fever and inflammation. However, there is no reported information on the antibacterial activities of *S. aurita* and *S. pyrolifolia*, and little is known about the phytochemistry of *S. aurita*. In this study, winter-dormant twig extracts of *S. aurita*, *S. caprea*, and *S. pyrolifolia* were screened for their antibacterial activities against *Pseudomonas aeruginosa*, *Staphylococcus aureus*, *Bacillus cereus*, and *Escherichia coli*. The antibacterial effects were evaluated using agar diffusion and turbidimetric microplate methods. Time-kill effects were measured using the microplate optical density (OD620) method. UPLC-PDA-QTOF/MS analysis was conducted to identify the polyphenols present in a methanol extract of *S. aurita*. The antibacterial results show that methanol and hot and cold water twig extracts of *S. aurita*, *S. caprea*, and *S. pyrolifolia* have significant antibacterial effects against *P. aeruginosa*, *S. aureus*, and *B. cereus* with the diameters of the inhibition zones (IZDs) ranging from 16.17 to 30.0 mm and the MICs between 1250 and 2500 µg/mL. Only the cold water extract of *S. caprea* was moderately active against *E. coli*. Proanthocyanidins, procyanidin B1 (*m*/*z* 577), and procyanidin C1 (*m*/*z* 865) were identified as the major polyphenols present in the methanol extract of *S. aurita* twigs for the first time. Additionally, salicin-7-sulfate was present in *S. aurita* twigs. Procyanidin B-1, taxifolin, trans-p-hydroxycinnamic acid, and catechin showed growth inhibitory activity against *B. cereus* with a MIC value of 250 µg/mL.

## 1. Introduction

The emergence of drug-resistant strains of bacteria due to the overuse, large availability, and overprescription of conventional antibiotics remains a global problem [1,2]. In recent times, the world is faced with the challenge to combat antimicrobial resistance (AMR), which poses a significant risk to human health. To date, infectious diseases caused by antibiotic-resistant bacteria are still one of the major causes of death globally [3,4]. The decreasing trend in the efficacy of these available conventional antibacterial drugs used for the treatment of common infections have given rise to the need for more effective antibiotics [5,6]. Thus, to minimize the public health crisis caused by multidrug-resistant bacteria, it is crucial to develop antibacterial drugs with a new mechanism of action to treat infectious diseases. Multidrug-resistant (MDR) bacteria, such as methicillin-resistant *Staphylococcus aureus* (MRSA), vancomycin-resistant Enterococci (VRE), and multidrug-resistant *Pseudomonas* (MDR PA), reduce the clinical efficacy of the newest approved antibacterial therapies, and numerous efforts to overcome resistance have not yielded optimum results [7]. Furthermore, research has shown that there is a current decline in antibacterial research and development, and this could pose a more catastrophic situation in medical care, and most especially in advanced medical treatment in the future [7,8]. It has been demonstrated in recent antimicrobial studies that plant-based secondary or special metabolites show promise in the search of new antibacterial compounds [9].

*Salix* extracts contain polyphenols such as phenolic acids, flavonoids, proanthocyanidins, and the salicinoids that are salicyl alcohol derivatives connected with a sugar moiety, which are biologically active compounds that may have a potential for drug discovery in pharmaceutical industries. Plant-derived natural compounds such as polyphenols are one of the sources of the chemical reservoirs utilized in the search of biologically active compounds for novel drug discovery [10,11,12,13,14]. Polyphenols are majorly classified according to their hydrocarbon-based aromatic rings into phenolic acids, stilbenes, lignans, flavonoids, hydrolysable, and condensed tannins, and they have been reported to have significant antibacterial activities [12,15,16]. The phenolic glucosides, salicortin, and salicin as well as the flavonoid, naringenin, have been identified among the biologically active compounds present in most *Salix* species [17,18]. Many phenolic glucosides that are specific to the plant family Salicaceae and predominantly present in *Salix* species have been reported to have anti-inflammatory, antirheumatic, antipyretic, and analgesic properties [18,19,20,21,22]. Moreover, 1,2-dihydroxybenzene or catechol, isolated from *Salix capensis* stem bark, gave promising antibacterial effects [23]. In addition, many species of the genus *Salix* are rich in condensed tannins, or proanthocyanidins. Procyanidin fractions obtained using Sephadex LH-20 fractionation have shown promising antibacterial effects [24]. Thus, to generate new antibacterial drug candidates or antibiotic adjuvants with new mechanisms of action, and thus, new efficacy, polyphenols from *Salix* spp., such as salicin and other salicinoids, procyanidins, flavonoids, and triterpenes, are tremendously valuable targets [20,25]. Thus, the increased knowledge of the antibacterial effects of the polyphenols in less studied *Salix* species, such as *S. aurita*, *S. caprea*, and *S. pyrolifolia* will be a crucial lead to the discovery of new antibacterial agents.

**Figure 1:**
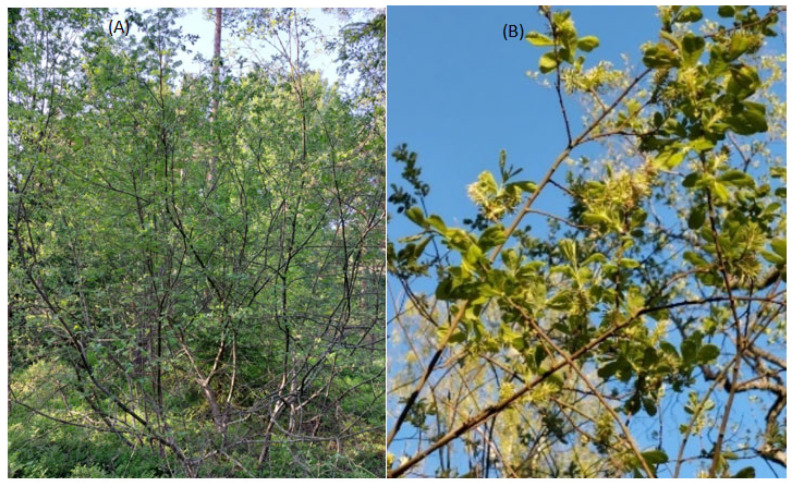
(**A**) A male *S. aurita* tree growing in a damp bog-like area in a mixed forest in Seurasaari, Helsinki. (**B**) A closeup of this tree, showing branches with the male catkins and new leaves in early June. (Photo credit: Pia Fyhrqvist).

*Salix* spp., also known as willows, are perennial, dioecious, woody plants comprising around 330–500 species occurring in Europe, Africa, Asia, and North America [19,26]. Extracts from *Salix* species have gained research interest over the years and decades due to their vast therapeutic use, health-promoting potentials, and their clinical evidence of safety with no serious side effects [11,27,28,29,30]. Traditionally, especially in Central and Southern Europe, willows are often collected from the wild and have been used in the treatment of fever, headaches, arthritis, rheumatism, and pain [31,32,33,34,35]. Also, the bark of willow species is prepared into powder and used as decoctions in ethnomedicine as well as recommended by the European Union Pharmacopeia as a 70% aqueous ethanol extract for the treatment of inflammation [36]. The efficacy of *Salix* extracts has been attributed to the presence of its pharmacologically active compounds such as salicin and other phenolic glucosides, flavonoids, and other polyphenols, including the proanthocyanidins [18,32,37]. *S. pyrolifolia* L., commonly called winter willow or pyrola-leaved willow, is a woody plant that has large and round leaves. It predominantly grows in nutrient-rich limestone regions along streams, in humid forests on slopes, and could be found only in three locations in the northern and eastern parts of Finland, where the populations consist of only female individuals [38]. However, it is easy to reproduce by cuttings. *S. aurita* L. is also known as the eared willow and is a shrub that is growing naturally in Finland and could grow between 1 and 3 m in height. *S. aurita* grows in wetlands, such as forest bogs and lake shores, but can also be found in forest and field margins, and it is one of the most abundant *Salix* species found in Finland [39] (Figure 1). *S. caprea* L., also called goat willow, is a deciduous shrub or tree that could grow between 10 and 15 m in height, and it is widely distributed across Europe and Asia. In Finland, goat willow is common in broadleaf and damp coniferous forests, shores, roadsides, and ditches. *S. caprea* has a fibrous root and old trees can have a much-branched trunk, which can reach considerable thickness. Moreover, *S. caprea* has greenish twigs with catkins, which appear between March and April before the leaves [40]. *S. caprea* is used in agriculture as a windbreak and its leaves are used as fodder for cattle and goats [40,41]. The extracts of the leaves, bark, and twigs of *S. caprea* are used in traditional medicine in the treatment of pain and inflammation, rheumatoid arthritis, malaria, and intestinal disorder [40,42]. The antibacterial activity of some *Salix* species, such as *S. myrsinifolia*, *S. cinerea* L., *S. caprea* L., *S. incana* Schrank, *S. rosmarinifolia* L., *S. sachalinensis* F. Schmidt, *S. acutifolia* L., *S. fragilis* L., *S. capensis* Thinb., and *S. caspica* Pall. has been reported [23,43,44,45]. Willow bark-derived fiber bundles also showed both antibacterial and antibiofilm activities against wound-isolated *Staphylococcus aureus* strains [46], while *S. alba* extracts were active against *Staphylococcus aureus*, *Streptococcus mutans*, and *Escherichia coli* [47]. However, to date, there is no reported information on the antibacterial activities of *S. aurita* and little is known about its phytochemistry and polyphenolic compounds. Apart from the studies by Julkunen-Tiitto [48,49], this study is among the few to report on the detailed phytochemical profiling of polyphenols, including procyanidins, from the twigs of *S. aurita*. Lavola et al. [38] reported on the presence of phenolic acid derivatives, phenolic glucosides, flavonoids, and condensed tannins from winter-dormant branches, including the wood, bark, and vegetative buds of *S. pyrolifolia*. However, apart from the reports by Julkunen-Tiitto [48,49] and Lavola et al. [38], there are few reports on the chemistry of *S. pyrolifolia*. It is noteworthy to mention that there is no report on the antibacterial activities of *S. pyrolifolia*. Therefore, the aim of this study is to investigate the antibacterial effects of the twig extracts of *S. aurita*, *S. caprea*, and *S. pyrolifolia*, and to identify the polyphenols present in an antibacterial twig methanol extract of *S. aurita*, which could be responsible for the antibacterial effects of this extract. Moreover, one of the aims of our study is to ascertain their reported use in traditional medicine as hot water decoctions and cold water macerations to treat gastrointestinal disorders and lung problems that could be of bacterial etiology [50]. Thus, this study will pave the way for further research on the antibacterial mechanism of actions of extracts, fractions, and polyphenols from *Salix* species as antibacterial agents.

## 2. Results

### 2.1. Extraction Yields of S. aurita, S. caprea, and S. pyrolifolia

**Figure 2:**
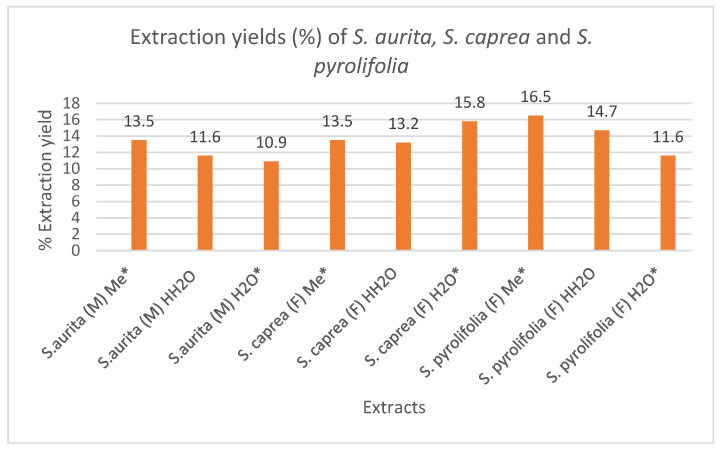
Extraction yields (%) of *S. aurita*, *S. caprea*, and *S. pyrolifolia*. Me*, cold methanol; HH_2_O, decoctions; H_2_O*, cold water extracts.

The results of the extraction yields (%) for the various twig extracts are shown in Figure 2. For each extraction solvent, the extraction was performed under the same extraction conditions and parameters for all the twig extracts and the yield were expressed as the dry matter content of each of the extracts. From the results of methanol extraction, the highest yield (16.5%) was recorded for *S. pyrolifolia*, followed by *S. caprea* and *S. aurita*, which resulted in extraction yields of 13.5% each, respectively. For the cold water extraction, the highest yield of 15.8% was recorded for *S. caprea*, followed by *S. pyrolifolia*, which had a 11.6% yield, and then *S. aurita*, which had a 10.9% yield. For the hot water extraction, *S. pyrolifolia* had the highest extraction yield (14.7%) while *S. caprea* had a 13.2% yield, and *S. aurita* had the lowest yield of 11.6%. From the results, it could be said that methanol and water (both hot and cold) are good extraction solvents for the *Salix* species used in this investigation.

### 2.2. Antibacterial Activity

A total of four clinically relevant Gram-positive (*S. aureus* and *B. cereus*) and Gram-negative (*P. aeruginosa* and *E. coli*) model human pathogenic bacterial strains were used to evaluate the antibacterial activity of the methanol and water extracts of the winter-dormant twigs of *S. aurita*, *S. pyrolifolia*, and *S. caprea*. In addition, several phenolic compounds that are present in these *Salix* spp. were screened. The results are presented below and shown in Table 1. The primary screening of the antibacterial effects of the twig extracts against the bacterial strains were performed using both the agar diffusion and microplate methods. In this screening, some of the results from the agar diffusion method correlated well with those from the microplate method. The extracts of *S. aurita*, *S. caprea*, and *S. pyrolifolia* were active against *P. aeruginosa*, *S. aureus*, and *B. cereus* when tested using the agar diffusion method. The diameters of the inhibition zones (IZDs) ranged from 16.0 to 30.0 mm (Table 1) and the minimum inhibitory concentrations (MICs) were mostly between 1250 and >2500 µg/mL, with a methanol extract of *S. aurita* giving the smallest MIC of 1250 µg/mL. *B. cereus* was the most sensitive bacterium to the extracts of *S aurita*, *S. caprea*, and *S. pyrolifolia* (Table 1). The methanol extract of *S. caprea* showed a large and remarkable clear inhibition zone of 30.0 mm and a promising growth inhibitory activity of 95.9% at 2500 µg/mL against *B. cereus*, which correlated well with the large size of the inhibition zone. Also, the large diameters of the zones of inhibition (30.0 and 29.8 mm) were observed with the cold water extract of *S. pyrolifolia* and a methanol extract of *S. caprea* against *S. aureus*, and these results correlated well with the microplate screening method where each of the extracts exhibited more than 90% growth inhibition at 2500 µg/mL (Table 1). The MIC values for a cold water extract of *S. pyrolifolia* and a methanol extract of *S. caprea* were both 2500 µg/mL against *S. aureus*. A large inhibition zone of 27.5 mm was also observed with *S. pyrolifolia* hot water extracts against *S. aureus*. However, it is interesting to note that while the hot water extract of *S. caprea* showed an inhibition zone of 22.5 mm against *S. aureus*, the cold water extract of *S. caprea* showed no inhibition against this bacterium. The cold water extract of *S. pyrolifolia* showed promising growth inhibitory activity against *B. cereus* with an inhibition zone diameter of 26.3 mm and a growth inhibition of 82.6% at 2500 µg/mL. The hot water extracts of *S. aurita* and *S. pyrolifolia* showed significant activity against *P. aeruginosa* with inhibition zone diameters of 20.8 mm and 22.0 mm, respectively. The MICs for *S. caprea* hot water and cold water extracts were 2500 µg/mL for both against *P. aeruginosa*. Moreover, the hot water extract of *S. caprea* was very active against *P. aeruginosa* with a 96% growth inhibition at 2500 µg/mL, and this result correlated well with the result obtained from the agar diffusion screening, which showed a 22.0 mm inhibition zone diameter. The MIC of the *S. aurita* extracts against *P. aeruginosa* were 2500 µg/mL for both the methanol, and hot water and cold water extracts. However, the MICs of some of the extracts, which were active in the agar diffusion and microplate screening methods, could not be reached when tested at 2500 µg/mL, as the percentage growth inhibition of these extracts at 2500 µg/mL was still under 90%. *E. coli* was resistant against most of the extracts of *S. aurita*, *S. caprea*, and *S. pyrolifolia* (Table 1). None of the methanol and hot water extracts of *S. caprea*, *S. aurita*, and *S. pyrolifolia* were active against *E. coli*. The extracts were devoid of antibacterial activity, both in the agar diffusion screening and in the microplate method. However, the cold water extracts of *S. caprea* and *S. aurita* were active against *E. coli* when tested with the agar diffusion method, showing inhibition zones of 22.0 mm and 18.3 mm, respectively. Furthermore, we studied the antibacterial effects of some commercial polyphenols such as salicortin, taxifolin, procyanidin B-1, trans-p-hydroxycinnamic acid, trans-m-hydroxycinnamic acid, catechin, acetylsalicortin, and others which are present in many *Salix* species. From the antibacterial results of the commercial compounds, it was observed that some compounds were more active than others (Table 1). Moreover, at the highest tested concentration of 500 µg/mL, the compounds showed just slight growth inhibitory activity against *P. aeruginosa*. Contrary to that, catechin and procyanidin B1 showed significant antibacterial activity against *B. cereus*, resulting in a growth inhibition of 98% and 95%, respectively, with a MIC value of 250 µg/mL. Notably, at 250 µg/mL, taxifolin and trans-p-hydroxycinnamic acid were very active against *B. cereus* and both showed a growth inhibition of 99%.

### 2.3. Time-Kill Effects of a Methanol Extract of S. aurita Against B. cereus and S. aureus

**Figure 3:**
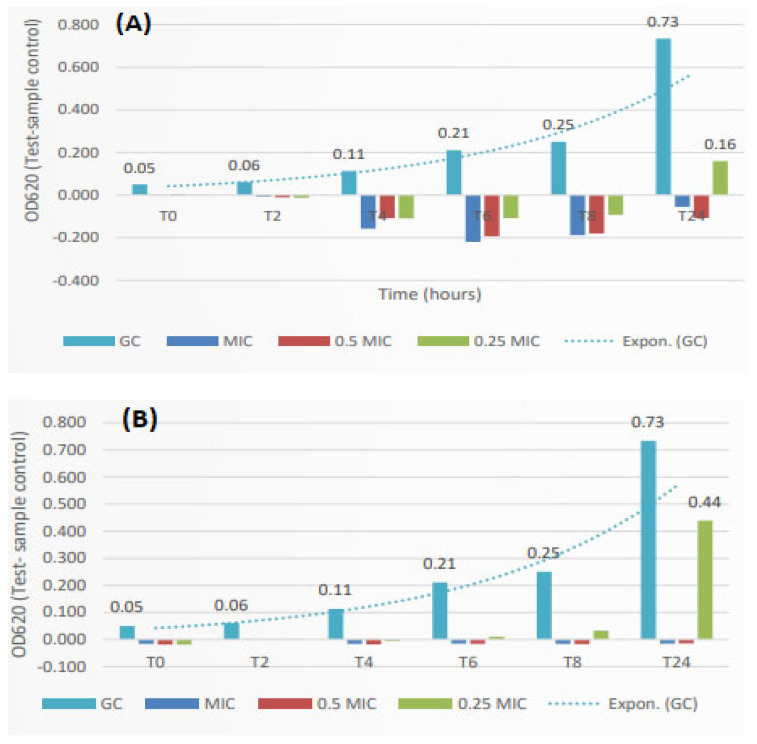
Time-kill effects of (**A**) a *Salix aurita* methanol extract and (**B**) tetracycline against *S. aureus*. The methanol twig extract was tested at MIC (1250 µg/mL), 0.5 × MIC (625 µg/mL), and 0.25 × MIC (312 µg/mL) concentrations. Tetracycline was tested at MIC (0.24 µg/mL), 0.5 × MIC (0.12 µg/mL), and 0.25 × MIC (0.06 µg/mL) concentrations. The turquoise bar indicates the growth control (GC).

**Figure 4:**
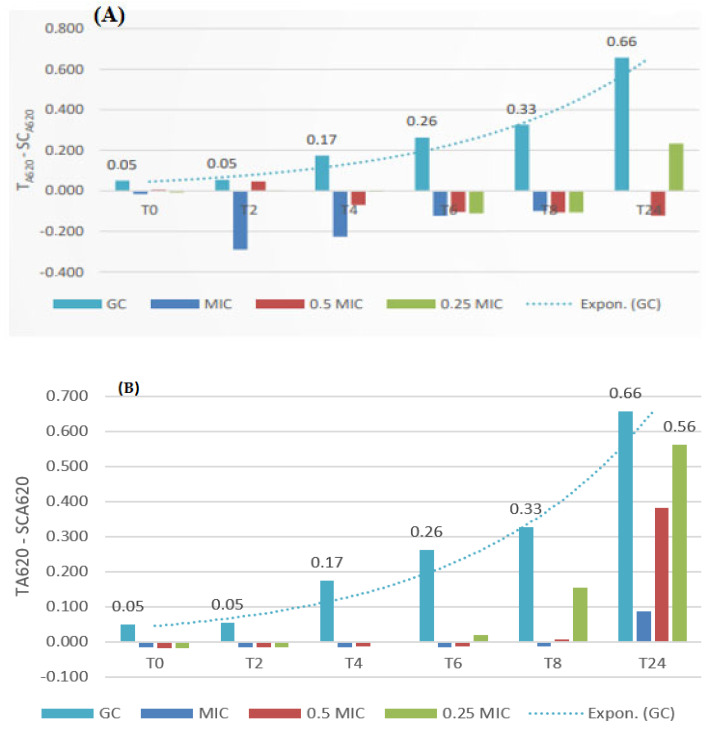
Time-kill effects of (**A**) a *Salix aurita* methanol extract and (**B**) tetracycline against *B. cereus*. The extract was tested at MIC (2500 µg/mL), 0.5 × MIC (1250 µg/mL), and 0.25 × MIC (625 µg/mL) concentrations. Tetracycline was tested at MIC (0.49 µg/mL), 0.5 × MIC (0.24 µg/mL), and 0.25 × MIC (0.12 µg/mL) concentrations. The turquoise bar indicates the growth control (GC).

The time-kill effects of the *S. aurita* methanol twig extract was monitored every second hour until 24 h during the growth of *S. aureus* and *B. cereus* to study the dynamic growth inhibitory effects (bacteriocidic or bacteriostatic) during the various phases of bacterial growth (Figure 3 and Figure 4). The *Salix aurita* methanol extract was chosen, since it exhibited good growth inhibition against *S. aureus* and *B. cereus* with MIC values of 1250 and 2500 µg/mL, respectively. The time-kill effects of the twig extract against *B. cereus* were measured using optical density (OD620). Figure 4A shows the growth kinetics in the time-kill assay of the methanol extract of the twigs of *S. aurita* against *B. cereus*. The extract at MIC (2500 µg/mL) and 0.5 × MIC (1250 µg/mL) totally inhibited the growth of this bacterium at all time-points until 24 h of incubation (Figure 4A). The extracts were also very active against the growth of *S. aureus* and the growth inhibition was total for the concentrations at MIC and 0.5 × MIC (1250 and 625 µg/mL, respectively) during all timepoints, with the exception of the smallest concentration at 312 µg/mL, which resulted in some bacterial growth after 24 h (Figure 3A). The time-kill effect of the *S. aurita* twig extracts was comparable to that of tetracycline, which was evaluated as a positive control against *S. aureus* and *B. cereus* at a concentration of MIC (0.24 µg/mL and 0.49 µg/mL, respectively), 0.5 × MIC (0.12 µg/mL and 0.24 µg/mL, respectively), and 0.25 MIC (0.06 µg/mL and 0.12 µg/mL, respectively) (Figure 3B and Figure 4B). The time-kill kinetics of this potent *S. aurita* extract against *B. cereus* and *S. aureus* shows that the extract at its MIC and 0.5 × MIC concentrations possesses bactericidal action against these bacteria and could be used as a topical agent against *S. aureus* skin infections.

### 2.4. UPLC/QTOF-MS Analyses for S. aurita

**Figure 5:**
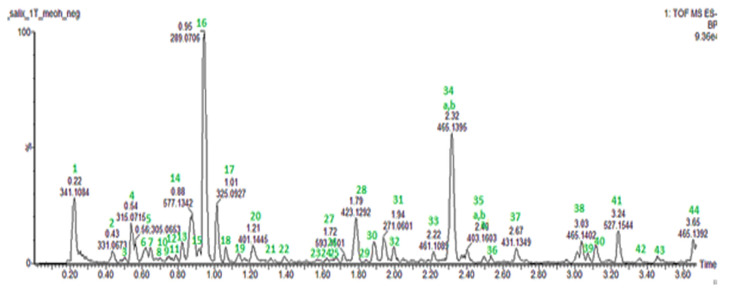
Total ion chromatogram (TIC) of identified polyphenolic compounds in an antibacterial methanol twig extract of *S. aurita*. The polyphenolic compounds that are numbered in green are also shown in Table 2.

**Figure 6:**
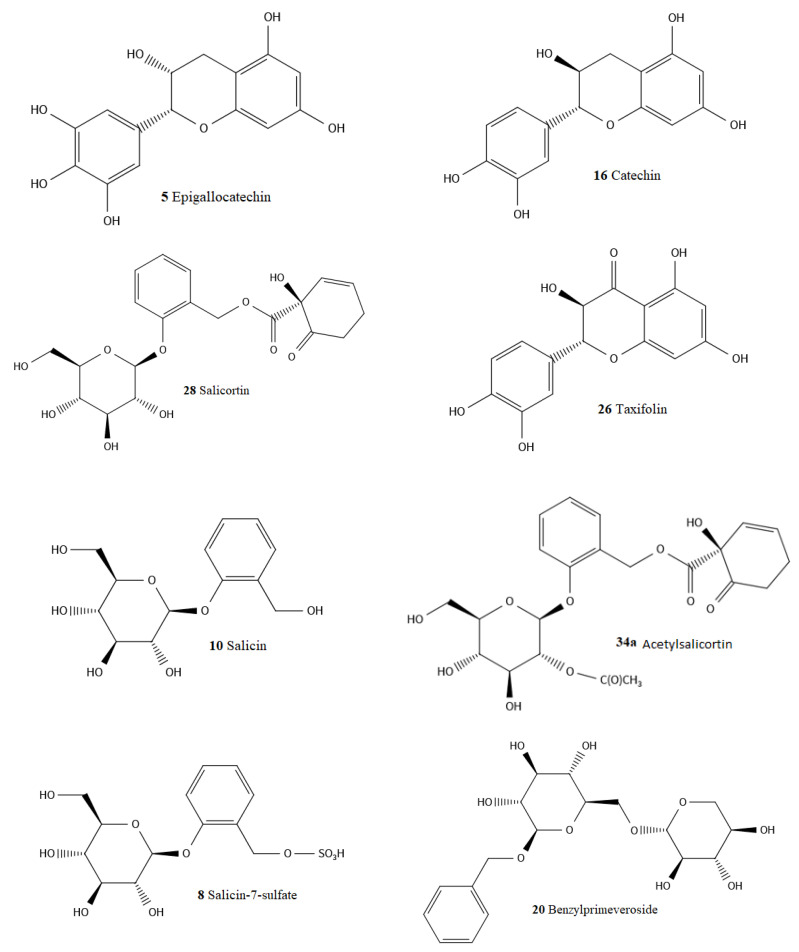

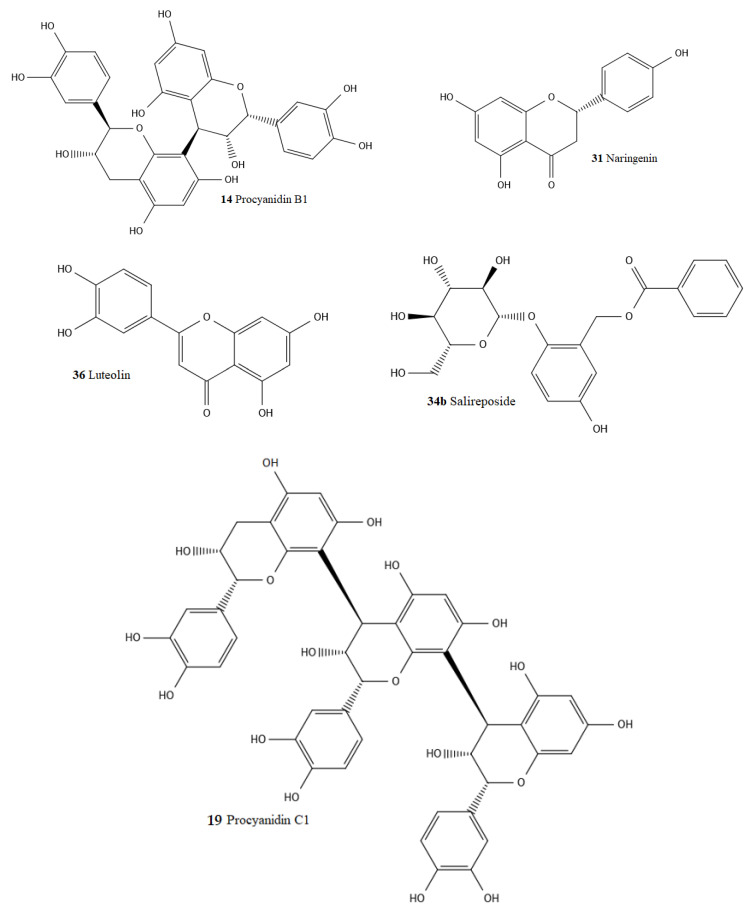
Chemical structures of some polyphenolic compounds identified in this study from an *S. aurita* methanol extract. The compounds are numbered as in Table 2.

**Figure 7:**
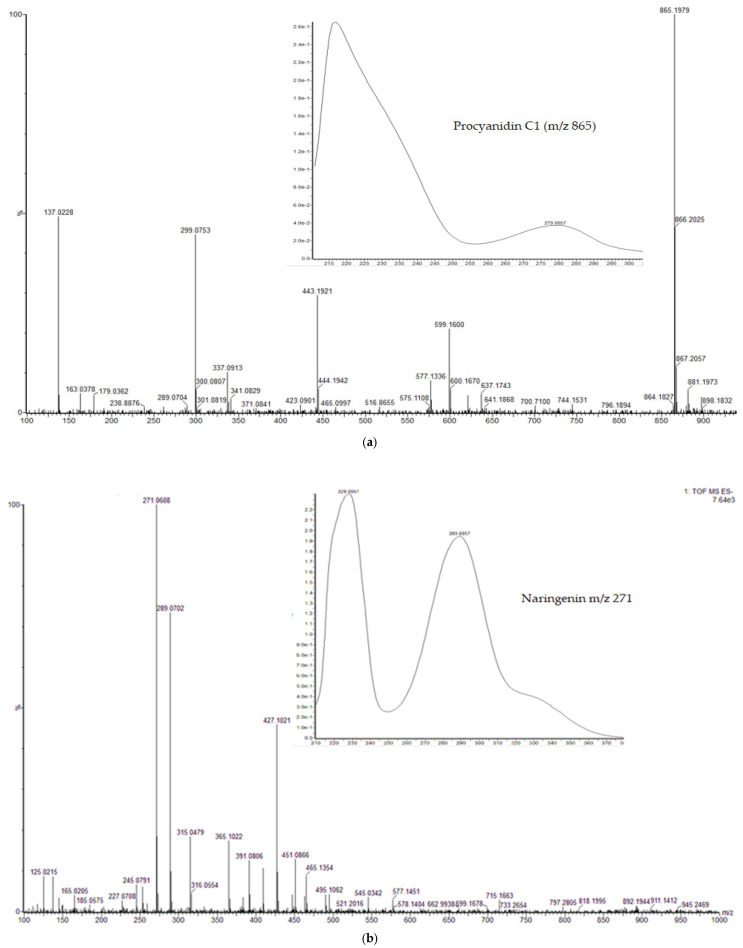

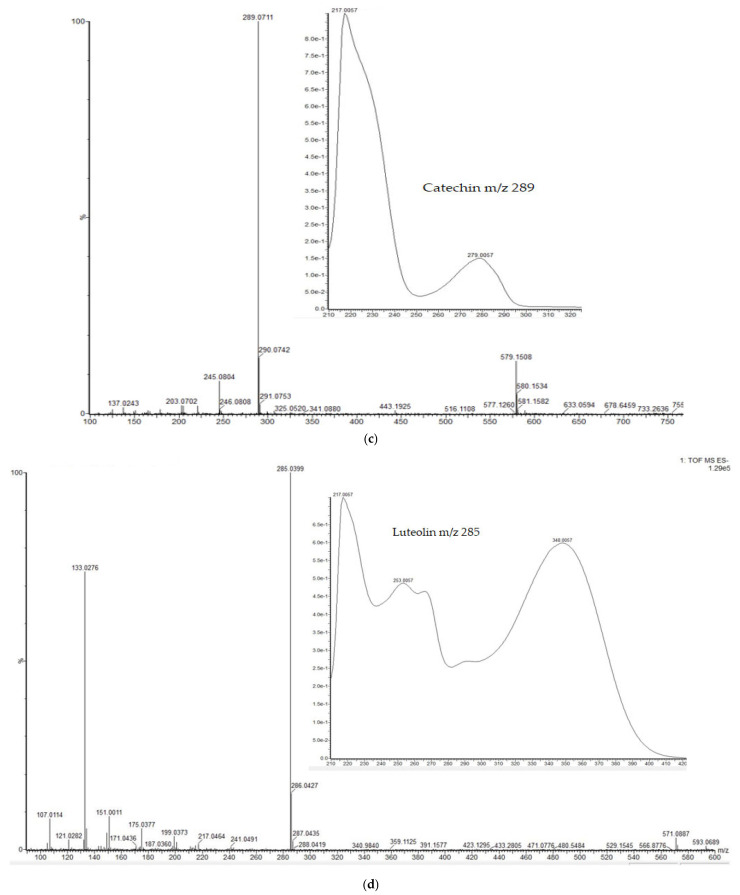
(**a**). Mass and UV absorption spectrum of procyanidin C1 from the methanol twig extract of *S. aurita*. Procyanidin C1 has a molecular ion [M-H]^−^ at *m*/*z* 865 and a chemical formula of C_45_H_38_O_18_. (**b**). Mass and UV absorption spectrum of naringenin from the methanol twig extract of *S. aurita*. Naringenin has a molecular ion [M-H]^−^ at 271 and a chemical formula of C_15_H_12_O_5_. (**c**). Mass and UV absorption spectrum of catechin from the methanol twig extract of *S. aurita*. Catechin has a molecular ion [M-H]^−^ at 289 and a chemical formula of C_15_H_14_O_6_. (**d**). Mass and UV absorption spectrum of luteolin from the methanol twig extract of *S. aurita*. Luteolin has a molecular ion [M-H]^−^ at 285 and a chemical formula of C_15_H_10_O_6_.

UPLC-PDA-QTOF-MS at negative electrospray ionization mode (ESI-) was used for phytochemical investigation and chemical profiling to identify the polyphenolic compounds present in a *S. aurita* methanol twig extract. In our study, we selected the *S. aurita* methanol extract of the twigs because this extract recorded the overall lowest MIC value of 1250 µg/mL against *S. aureus* and was significantly active against most of the tested bacteria, including *P. aeruginosa* and *B. cereus*. Moreover, there exists no detailed study on the polyphenol composition of the twigs of *S. aurita*. From the UPLC-PDA-QTOF-MS analysis, a total of 44 polyphenolic compounds, including several salicylates, the main phenolic glucosides in *Salix* spp., as well as flavonoids, phenolic acids, and procyanidins were identified in *S. aurita* (Table 2 and Figure 5). Also, a mixture of standard commercial compounds found in *Salix* species such as luteolin, catechin, salicin, taxifolin, salicortin, naringenin, acetylsalicortin, and procyanidin-B were evaluated for their retention times, UV absorption spectra, and molecular ions. The chemical identification of the compounds in *S. aurita* was achieved by comparing the retention times of the eluted compounds, their molecular ions (*m*/*z*) in negative ionization mode, their UV absorption spectra with those of the commercial reference standards, and with data from the previous literature [38,48,49]. The most predominant compounds in the extracts of *S. aurita* were various catechin derivatives, catechin, proanthocyanidins (condensed tannins), and phenolic glucosides (Figure 6). Some of the polyphenolic compounds identified in the extract were previously known in *S. aurita* and in other *Salix* species. For example, among the polyphenolic compounds identified in *S. aurita* extract, salicortin (**28**) with a retention time of 1.79 min and a molecular ion [M-H]^−^ at *m*/*z* 423.1291, and salicin (**10**) with a retention time of 0.76 min and a molecular ion [M-H]^−^ at *m*/*z* 285.0977, were previously known to be present in *S. aurita* [48,49]. However, salicin-7-sulfate (**8**) with a retention time of 0.68 min and a molecular ion [M-H]^−^ at *m*/*z* 365.0542 was identified for the first time in *S. aurita*. Procyanidin B1 (**14**) with a molecular ion [M-H]^−^ at *m*/*z* 577.1342, which is a proanthocyanidin, was detected at a retention time of 0.88 min. Naringenin (**31**) with a molecular ion [M-H]^−^ at *m*/*z* 271.0606 was detected at a retention time of 1.94 min, and taxifolin (**26**) with a molecular ion [M-H]^−^ at *m*/*z* 303.0505 had a retention time of 1.69 min. Moreover, catechin (**16**) with a retention time of 0.95 min, showing a molecular ion [M-H]^−^ at *m*/*z* 289.0712, and luteolin (**36**) with a retention time of 2.54 min, showing a molecular ion [M-H]^−^ at *m*/*z* 285.0399 were detected in the extract. The other polyphenolic compounds found in the extract were the catechin–gallocatechin isomers with a molecular ion [M-H]^−^ at *m*/*z* 593.1294 (**3**, **6,** and **13**). Procyanidin C1 (**19**) was also identified with a molecular ion [M-H]^−^ at 865.1968 and a retention time of 1.14 min. The mass and UV spectra of procyanidin C1, naringenin, catechin, and luteolin are shown in Figure 7a–d. Our results also revealed protocatechuoylglucose (**4**) at Rt 0.54 min with a molecular ion [M-H]^−^ at 315.0714.

## 3. Discussion

In this study, the twig extracts of *S. aurita*, *S. caprea*, and *S. pyrolifolia* collected from the Helsinki, Luikonlahti, and Lammi regions in Finland have revealed significant antibacterial activity against both Gram-positive and Gram-negative bacterial strains. Also, some of the commercial pure compounds, including taxifolin, procyanidin B1, and catechin that we have found to be present in the studied *Salix* species have shown remarkable and potent antibacterial activities, especially against the Gram-positive *Bacillus cereus*. In this study, two pathogenic Gram-negative bacterial strains (*P. aeruginosa* and *E. coli*) were chosen for antibacterial screening because they easily develop resistance to antibiotics, and to date, the threats caused by these Gram-negative bacteria is of increasing global healthcare concern [51,52]. *P. aeruginosa* is a bacterium that causes serious opportunistic infections in humans, most especially in immunocompromised individuals with acquired immunodeficiency syndrome (AIDS), severe burns, and cancer [53]. *P. aeruginosa* can cause both acute and chronic infections in immunocompetent and immunocompromised individuals, thereby leading to high morbidity and mortality [51,54]. Multidrug-resistant (MDR) and extensively drug-resistant (XDR) lineages of *P. aeruginosa* infections are common in wound and burn patients all over the world and only a few antibiotics are effective in the treatment [51,55]. For the Gram-positive bacteria, *S. aureus* and *B. cereus* were chosen for the study because of their clinical importance. Methicillin-resistant *Staphylococcus aureus* (MRSA) is of concern in public health, rural communities, and in healthcare facilities [56,57]. Significantly, it has been reported that *E. coli* is the second leading pathogen causing neonatal meningitis [51]. Furthermore, plant-derived natural products and plant extracts, including *Salix* species, could be used as sources of new antibacterial compounds against multidrug-resistant (MDR) pathogens, including methicillin-resistant *Staphylococcus aureus* (MRSA) [58,59]. From our study, the overall most significant antibacterial activities were observed with the Gram-positive bacteria as we recorded large diameters of inhibition zones ranging from 20.0 to 30.0 mm and MIC values ranging from 1250 to 2500 µg/mL. For example, the largest inhibition zone of 30.0 mm was observed with the cold water extract of *S. pyrolifolia* against *S. aureus* and this correlated well with the growth inhibition of 92% and a MIC value of 2500 µg/mL of this extract. A large inhibition zone of 27.5 mm was also observed with *S. pyrolifolia* hot water extracts against *S. aureus*. Thus, especially cold water macerations, but also hot water extracts of *S. pyrolifolia*, could have value as standardized extracts in traditional medicine to treat common foodborne bacterial infections caused by *S. aureus* or to prevent bacterial contamination in foods, for example, as an ingredient in antibacterial food packages. Our result justifies the reported use of *Salix* extracts in traditional medicine to treat infections [19]. Also, a 30.0 mm inhibition zone was observed with the methanol extract of *S. caprea* against *B. cereus*, and this correlated well with the growth inhibition of 96% when tested at 2500 µg/mL. The good antibacterial activity recorded with the methanol extracts of *S. caprea* against *B. cereus* indicates that methanol could be a good solvent for extracting compounds active against *B. cereus.* These results demonstrate that when compared to the water extracts, the *S. caprea* twig methanol extract contains either more antibacterial compounds or different compounds with specific activity against *B. cereus*. In addition, and very interestingly, water is also a good extracting solvent for most of the *Salix* species in this present study, justifying the traditional use of *Salix* species water extracts for the treatment of gastrointestinal infections [40,42]. However, in some cases, the decoctions were found to be more active than the cold water extracts, and vice versa. This indicates that cold and hot water extracts might contain compounds in different proportions and even partly different compounds (more lipophilic compounds could be extracted in the hot water).

The lowest MIC value of 1250 µg/mL for this antibacterial study was observed with a methanol extract of *S. aurita* twigs against *S. aureus.* Moreover, this extract also showed a large inhibition zone diameter of 23.8 mm against *S. aureus*. All the *S. aurita* extracts (methanol, hot water, and cold water) were significantly active against *S. aureus.* Additionally, both the cold and hot water extracts also showed good activity against *P. aeruginosa*. In addition, the *S. aurita* methanol extract showed good activity against *B. cereus*. Our time-kill study indicates that the *S. aurita* methanol extract is bacteriocidic at its MIC (2500 and 1250 µg/mL, respectively) and 0.5 × MIC concentrations against both *B. cereus* and *S. aureus*, allowing no bacterial growth even after a 24 h incubation time. To the best of our knowledge, this is the first report on the antibacterial effects of a *S. aurita* twig extract and our study suggests that *S. aurita* extracts may contain new antibacterial agents which could be explored as new lead compounds to combat infections caused by *S. aureus* and *B. cereus*. Our results agree with the time-kill results of Mai et al. [60], who found that a methanol leaf extract of *Salix babylonica* at its 2 × MIC concentration (4 mg/mL) showed significant growth inhibitory effects throughout a 24 h incubation time of *Vibrio parahaemolyticus*. Additionally, in agreement with our study, yarn containing at least 50% of willow bark fiber bundles from the *Salix* variety called Klara (with *Salix dasyclados*, *S. schwerinii*, and *S. viminalis* as the parent species of this variety) significantly inhibited biofilm formation by clinically isolated biofilm-forming wound-isolated *S. aureus* strains, and it was reported that polyphenols, such as condensed tannins present in the willow bark-derived material, were largely responsible for the antibacterial and antibiofilm activities [46]. In another study, willow bark-derived material showed a remarkable antibacterial activity against a laboratory strain of *S. aureus*, which resulted in the complete eradication of the viable bacteria after 24 h of incubation with the willow bark-derived material [61]. This result agrees with our results that a methanol twig extract of *S. aurita* at its MIC and 0.5 × MIC concentrations completely eradicated the growth of *S. aureus* and *B. cereus* after a 24 h incubation.

In our study, we observed the potent antibacterial effects of a *S. caprea* methanol extract against *S. aureus* with inhibition zones of 29.8 mm. Also, and significantly, our screening results revealed that the cold water extract of *S. caprea* was very active against *P. aeruginosa* with a growth inhibition of 96% at 2500 µg/mL, and this result correlated well with our result obtained from the agar diffusion screening, which showed a 22.0 mm inhibition zone diameter. Our results complement a previous study which demonstrates that the lipophilic extracts of *S. caprea* are growth inhibitory against *S. aureus* and *P. aeruginosa*, with inhibition zones of 31.1 and 26.2 mm, respectively [45]. These results open the way for more research into fractions and compounds in the *S. caprea* extracts of various polarities as sources of antimicrobial compounds to combat infections caused by *P. aeruginosa*.

In our antibacterial screening using *E. coli*, only the cold water extracts of *S. caprea* and *S. aurita* were moderately active with inhibition zones of 22.0 mm and 18.3 mm, respectively. The rest of the extracts that we screened were not active against *E. coli*. This is an indication that the cold water extracts may contain antibacterial compounds, which may be useful to combat *E. coli* strains, but more research is needed. In agreement with our findings that most of the *Salix* twig extracts were not active against *E. coli*, Jang et al. [30] reported that all the marker compounds isolated from the ethanolic extracts of *S. caprea* and other *Salix* species, including 2′-*O*-acetylsalicortin and salicin, did not show antibacterial activity against *E. coli.* However, in accordance with our results that the water extracts of *S. aurita* and *S. caprea* inhibit *E. coli*, Tienaho et al. [62] reported that water extracts of willow bark clones were active against *E. coli*. Therefore, the water extracts of *Salix* spp. might differ from ethanolic extracts in their chemical composition, containing a higher number of compounds that are particularly inhibitory against *E. coli*. Water-soluble procyanidins, which occur in abundance especially in the bark of studied *Salix* species, could play an important role in inhibiting the growth of *E. coli* [63]. The antibacterial and time-kill efficacy of polyphenol-rich extracts and polyphenols are affected by the method of extraction, the cell structure of the bacterium, the inoculum density, the treatment time, and the level of exposure of the polyphenol or polyphenol-rich extract on the bacterium. To strengthen the accuracy of our time-kill results and to ascertain the antibacterial efficacy during the various phases of bacterial growth, we took into consideration all these factors. Various proanthocyanidins, flavonoids, and phenolic glycosides could be responsible for the significant antibacterial activity that was observed in our study with *S. aurita* methanol, and cold and hot water twig extracts. The result from our study agrees with previous findings that proanthocyanidins and gallate esters consisting of procyanidins, catechin, and epicatechin units showed potent antibacterial activities against 10 different Gram-positive and Gram-negative pathogenic bacterial strains of clinical importance [24].

UPLC/QTOF-MS, which is one of the major analytical techniques used to identify and generate the chemical profile of secondary metabolites in plant extracts, were used for the phytochemical and structural investigation. From the results of our phytochemical investigation, the *S. aurita* methanol extract was rich in polyphenolic compounds, and some of the compounds, such as the procyanidins, have been reported by previous studies to exhibit antibacterial activities against numerous bacterial strains [10,64]. The *S. aurita* extract was found to contain mostly proanthocyanidins or condensed tannins, which are the oligomeric forms of flavan-3-ols. Proanthocyanidins have been reported to be one of the most significant target compounds from natural sources in antibacterial drug discovery [24,65]. In our study, procyanidin B1 (**14**), which we found as one of the major compounds in *S. aurita* twigs, showed significant antibacterial activity against both *Bacillus cereus* and *Pseudomonas aeruginosa*. Moreover, we discovered the occurrence of procyanidin C1 (**19**) in *S. aurita* twigs. Procyanidin B1 and procyanidin C1, which are composed of (+)-catechin and (−)-epicatechin structural units, have been previously reported to have bacteriostatic effects against *S. aureus* and other bacterial strains [66,67]. The procyanidin B1 (**14**), with a molecular ion [M-H]^−^ at *m*/*z* 577, and procyanidin C1 (**19**), with a molecular ion [M-H]^−^ at 865, that we identified in *S. aurita* have previously been found to be the most abundant phenolic polymers present in high concentrations in *Salix fragilis* and *Salix viminalis* [68]. However, our study is the first report on the identification of procyanidin B1 and C1 in *S. aurita*. Moreover, by using the butanol-HCL assay for the quantitative estimation of the tannin content in *S. aurita*, Julkunen-Tiitto [50] showed that this species contains moderate concentrations of condensed tannins in accordance with our results. In this present study, we identified several other procyanidins in the *S. aurita* twig extract, including the polyphenolic compounds (**3**, **6,** and **13**) with a molecular ion [M-H]^−^ at *m*/*z* 593, which were identified as catechin–gallocatechin isomers that have been previously identified in the leaf and bark extracts of *S. alba* [69]. Procyanidins have been reported to be very active against most bacterial strains [66]. More so, procyanidin-rich grape seed extract was found to have potent inhibition against the biofilm formation of *E. coli* [70].

We found that catechin (**16**) was largely present in the twigs of *S. aurita* and our study agrees with a recent study that catechin is the most predominant flavan-3-ol occurring at the highest concentration in *S. daphnoides*, *S. fragilis*, *S. dasyclados*, *S. viminalis*, and *S. dasyclados × viminalis* [68]. Previous research has shown that catechin and its derivatives actively inhibited the growth and biofilm formation of three methicillin-resistant *S. aureus* (MRSA) strains in a dose-dependent manner [71].

In this study, we identified naringenin (**31**) in the twigs of *S. aurita*. Naringenin was previously found to be present in *S. pyrolifolia*, *S. daphnoides*, *S. fragilis*, *S. dasyclados*, and *S. viminalis* [38,67]. Naringenin, which is a natural flavonoid found in plants, has been reported to have antibacterial activity against *S. aureus* and *E. coli* [72]. Salicin (**10**) is present in *S. aurita* twigs and is known to occur in small concentrations in most willow species. Salicin showed antibacterial activity against *S. aureus* in vitro and a mouse in vivo pneumonia model, which showed that salicin reduces the virulence of *S. aureus* [73]. Furthermore, galloylglucose (**2**), protocatechuoylglucose (**4**), and salicortin (**28**), which were identified in our methanol extract of *S. aurita* twigs and in a methanol extract of *Salix tetrasperma* stem bark, have been reported to inhibit the quorum sensing of *P. aeruginosa*. Moreover, the mentioned compounds inhibit the proteolytic and hemolytic activities of *P. aeruginosa* in a dose-dependent manner, of which the latter mentioned activities are related to the bacterial ability to invade host tissues [43]. Taxifolin (**26**), catechin (**16**), and procyanidin B1 (**14**), which have been found to be present in many *Salix* species, and in this present investigation, in the twigs of *S. aurita*, could be responsible for the remarkable antibacterial activity of the *Salix* extracts against *B. cereus*.

It is important to note that the Finnish weather conditions could affect the content of these bioactive compounds since previous research has shown that phenolic contents are often affected by the environment and weather conditions [74]. In a study by Förster et al. [32], it was found that the secondary metabolites concentration in willow bark decreased during the vegetation period from March to July. Additionally, Dou et al. [75] showed that the composition of sugars in willow were dependent on the season. In this present study, the winter-dormant twig material of *Salix aurita*, *S. caprea*, and *S. pyrolifolia* were used for the antibacterial screenings and our screening results could be compared to plant material collected during the growing season and fall.

## 4. Materials and Methods

### 4.1. Collection of Plant Materials

**Figure 8:**
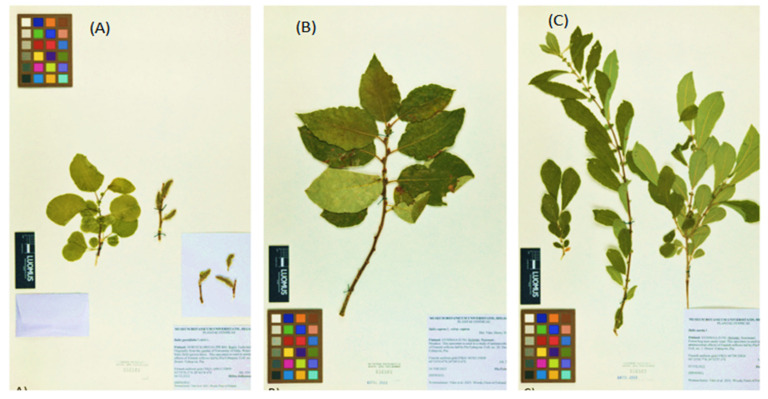
Voucher specimens of (**A**) *Salix pyrolifolia*, (**B**) *S. caprea*, and (**C**) *S. aurita* used in this investigation as deposited at the herbarium of the Botanical Museum, University of Helsinki, Finland. Photo credit: Jaana Haapala.

The winter-dormant twigs of *S. aurita* and *S. caprea* used in the study were collected in February and April in 2022 during the winter season from Lammi and Helsinki in Southern Finland, while the twigs of *S. pyrolifolia* were from a cultivated female clone collected in March 2022 from Luikonlahti in Eastern Finland. The winter-dormant twigs were chosen because they have been previously reported to be rich and more abundant in polyphenolic compounds compared to summer twigs [32,38], and this could have a significant effect on the antibacterial activity. Each of the twig samples were labelled with cable ties and given a code for proper identification and for the purpose of re-sampling. The identity of the twig samples was authenticated and confirmed based on summer twigs with fully developed leaves from the collected *Salix* specimen by an expert in botany and taxonomy, Dr. Henry Väre, Curator of the Botanical Museum, Finnish Museum of Natural History, University of Helsinki, Finland. Voucher specimens were prepared and deposited at the herbarium of the Botanical Museum, University of Helsinki, Finland (Figure 8). The collected twigs were spread in the laboratory table for three weeks to dry and then cut into smaller pieces for easy grinding. The dried twig samples were milled using a grinding machine and labelled appropriately, ready for extraction.

### 4.2. Extraction

#### 4.2.1. Decoctions and Macerations

Decoctions and macerations of the *Salix* species were prepared as in Mgbeahuruike et al. [76]. For the decoctions (hot water extraction), 5 g of the dried and milled twig samples were weighed into a 500 mL *v*/*v* Erlenmeyer flask (Darmstadt, Germany) and 150–200 mL of distilled water was added. The twig samples were brought to a boil for 5 min and then the extraction was continued overnight using a magnetic stirrer (Fisher, Italy). For the maceration (cold water extraction), the same method was used except that the mixture was not brought to a boil for 5 min. After 24 h, the extracts were decanted and the liquid part was collected into centrifuge tubes (Eppendorf 5810 R, volume 50 mL, Hamburg, Germany) and centrifuged at 3000 rpm for 10 min at the temperature of 22 °C. The supernatants from the centrifuge tubes were carefully collected into a beaker, labelled properly, covered with perforated parafilm, and stored at −20 °C. The frozen filtrates were then lyophilized for two days to completely freeze dry using SCANVAC Cool safe 110-4 Pro lyophilizer (Labogene, Denmark). The freeze-dried extracts were later re-dissolved in methanol to stock solutions at 50 mg/mL that were used for the antibacterial screenings.

#### 4.2.2. Methanol Extraction

A total of 20 g of the air-dried and milled plant material was carefully weighed, and 500 mL of 100% methanol was added to the extracts in an Erlenmeyer flask (Darmstadt, Germany). Extraction was performed overnight using a magnetic stirrer. After 24 h, the mixture was filtered using the filtration Büchner funnel kit vacuum suction glass flask apparatus and Whatman filter paper (Schleicher & Schuell, ø 150 mm, Dassel, Germany). The filtrates were collected and transferred into a round bottomed flask of known weight and then the methanol was evaporated using a rotary evaporator apparatus (Rotavapor, Heidolph VV2000) combined with a water bath set at a temperature of 40 °C. The extracts were labelled properly, covered with perforated parafilm, and stored at −20 °C. The frozen filtrates were then lyophilized for two days to completely freeze dry using a SCANVAC Coolsafe 110-4 Pro lyophilizer (Labogene, Denmark). The percentage extraction yield of the twig extracts was calculated using the formula:

(1) $$Extraction\;yield\left(\%\right)=\frac{Weight\;of\;dry\;twig\;extract}{Weight\;of\;dry\;twig\;sample\;before\;extraction}\times 100$$

### 4.3. Bacterial Strains and Antibacterial Assay

#### 4.3.1. Bacterial Strains, Commercial Compounds, and Antibiotics

The in vitro growth inhibitory activity of the twig extracts was evaluated using *Staphylococcus aureus* ATCC 25923, *Bacillus cereus* ATCC 10987, *Escherichia coli* ATCC P25922, and *Pseudomonas aeruginosa* ATCC 27853. Some commercially available compounds identified in *Salix* species were screened for their antibacterial activity. Analytical grade naringenin (Biopurify Chemicals Ltd., Chengdu, China), procyanidin B1 (22411, Cayman, Ann Arbor, MI, USA), taxifolin (22411, Cayman, Ann Arbor, MI, USA), catechin (Biopurify Chemicals Ltd., Chengdu, China), salicortin (Sigma-Aldrich, Darmstadt, Germany), trans-p-hydroxycinnamic acid (Sigma-Aldrich), trans-m-hydroxycinnamic acid (Sigma-Aldrich), salicin and acetyl-salicortin (Sigma-Aldrich) were the pure compounds screened. Gentamicin (Sigma-Aldrich, St. Louis, MO, USA), tetracycline hydrochloride (Sigma-Aldrich, St. Louis, MO, USA), rifampicin (Sigma-Aldrich, St. Louis, MO, USA), and penicillin (Sigma-Aldrich, St. Louis, MO, USA) were used as standard antibiotics for the investigation.

#### 4.3.2. Agar Diffusion Assay

An agar diffusion method was used as a reference method to screen the antibacterial activity of the polar twig extracts of *S. aurita*, *S. caprea*, and *S. pyrolifolia* as described by Mgbeahuruike et al. [76]. The freeze-dried extracts were re-dissolved in methanol and a stock solution of 50 mg/mL concentration was prepared for the extracts. Each of the pure compounds as well as the antibiotics, which were used as positive controls in the antibacterial testing, were prepared to a final concentration of 10 mg/mL in methanol. The petri dishes used for the screening were sterile petri dishes (ø = 15 cm, VWR International Oy, Helsinki, Finland). The petri dishes were prepared by adding 26 mL of sterile base agar (Antibiotic agar No. 2, Difco, VWR) as a bottom layer using a sterile serological pipet (Falcon; BDLabware Europe) and allowing it to stay for few minutes to solidify, and then, 26 mL of isosensitest agar (OXOID, ThermoFisher Scientific, Waltham, MA, USA) was applied as the top layer. The agar in the petri dishes was allowed to stay for a few minutes to completely solidify and the petri dishes were properly labelled and then stored at +4 °C. The bacterial strains were recovered from the −80 °C cryopreservation and were grown overnight for 24 h on nutrient agar slants at +37 °C before the test. A few colonies of the bacteria were taken from the nutrient agar slants using an inoculation loop and transferred to 2 mL of Mueller–Hinton broth or 0.9% NaCl solution in a sterile glass tube, which was shaken vigorously thereafter the suspension was divided into two parts: 1 mL of the bacterial suspension was transferred into another sterile glass tube, and 1 mL was pipetted into a cuvette to measure the optical density at 625 nm (UV–Visible Spectrophotometer, Pharmacia LKB-Biochrom 4060). From the results of the turbidimetric measurement, the remaining 1 mL in the sterile glass tube was diluted to an OD of 0.1 at 625 nm (containing approximately 1.0 × 10^8^ CFU/mL) with Mueller–Hinton broth or 0.9% NaCl solution. A total of 200 µL of the inoculum was evenly spread on each of the petri dish. The petri dishes were allowed to stay for a few seconds to dry with the lids kept open. A sterile cork borer (12 mm in diameter) was used to make six holes equidistantly from each other on the agar surface in the petri dishes. A total of 200 µL of the plant extracts (50 mg/mL) and 200 µL of the antibiotics and pure compounds (10 mg/mL) were carefully pipetted into the holes on the agar surface. The petri dishes were kept in the cold room at +4 °C for 1 h before incubation, after which the petri dishes were incubated overnight for 24 h at +37 °C. After 24 h of incubation, the diameters of the zones of inhibition were measured with a caliper under a petri dish magnifier and expressed as the mean of the diameters of three replicates ± SEM.

#### 4.3.3. Screening at One Starting Concentration Using a Turbidimetric Microplate Assay

In addition to the agar diffusion method, a turbidimetric microplate method was used for the primary screening to evaluate if the *Salix* extracts were active at a starting concentration of 2500 µg/mL. Moreover, a starting concentration of 500 µg/mL was used for the pure compounds. Before the test, a few colonies were transferred to 20 mL of nutrient broth and grown overnight for 24 h at +37 °C with shaking at 200 RPM in an orbital incubator (Stuart^®^ SI500289, London, UK). To prepare the inoculum, 2 mL of the overnight bacterial culture was taken, and divided between two test tubes, with 1 mL in each. The absorbance or optical density (OD) of 1 mL of the overnight bacterial culture was measured at 625 nm, using a UV–visible spectrophotometer (Pharmacia LKB-Biochrom 4060; Pfizer Inc., New York, NY, USA). According to the OD_625_ result, the suspension in the other test tube was diluted with Mueller–Hinton broth to reach an absorbance of 0.1 at 625 nm (containing approximately 1.0 × 10^8^ CFU/mL). Furthermore, a 100-fold dilution was prepared by taking 100 µL from the diluted A625 = 0.1 suspension and further diluting it in 9.9 mL of Mueller–Hinton broth to obtain the inoculum containing 1 × 10^6^ CFU/mL, as recommended by the Clinical Laboratory Standards Institute [77]. A total of 100 µL of this inoculum, and 100 µL of the plant extracts (5 mg/mL), pure compounds (1 mg/mL), and antibiotics (1 mg/mL) were introduced into the 96 microwell plates. The solvent control, MeOH, was not toxic at a 5% (*v*/*v*) volume in the wells, which was the maximum volume of this solvent used. The growth control (GC wells) contained only the bacterial suspension, and the test wells (T wells) contained plant extracts or pure compounds + bacterial suspension, while the negative control wells (NC wells) contained plant extracts or pure compounds and broth. These negative control wells were prepared for each plant extract/compound to be tested to subtract the light absorbance of extracts and compounds from the wells containing the corresponding extracts/compounds with bacteria. The microwell plates were incubated for 24 h in a BioSan incubator (Thermo-Shaker PST-60HL-4) at +37 °C, 350 RPM. The turbidity of the wells at 620 nm was recorded using a Multiskan Sky Microplate Spectrophotometer (Thermo Fisher Scientific) and the antibacterial activity was subsequently calculated as expressed as the mean percentage growth inhibition of duplicate wells.

#### 4.3.4. Minimum Inhibitory Concentration (MIC) Evaluation Using a Turbidimetric Microdilution Method

From the results obtained from the primary antibacterial screening of the twig extracts of *S. aurita*, *S. caprea*, *S. pyrolifolia*, pure compounds, and antibiotics, the minimum inhibitory concentration (MIC) was evaluated for some selected extracts, antibiotics, and pure compounds showing promising growth inhibition at one starting concentration. A microdilution turbidimetric broth method was used as outlined by the Clinical and Laboratory Standards Institute [77]. In short, the same method was used as described above, but instead of one concentration, two-fold dilutions of the plant extracts, pure compounds, and antibiotics were tested. Furthermore, only extracts and compounds with the most significant antibacterial activity in the screenings at 2500 and 500 µg/mL, respectively, were chosen for the MIC evaluation. The aim of the MIC screening was to find the lowest concentration of the twig extracts, pure compounds, or antibiotics that could inhibit at least 90% of the growth of the bacterial strains (which in practice means that there is no visible growth). For the MIC evaluation, the *Salix* twig extract stock solutions (50 mg/mL) were first diluted 10-fold with sterile Mueller–Hinton broth to obtain a 5 mg/mL concentration. Two-fold serial dilutions were subsequently prepared in Eppendorf tubes (2 mL volume) starting from 2500 to 19 µg/mL using sterile Mueller–Hinton broth. For the pure compounds identified in *Salix* species and the antibiotics, a 1 mg/mL concentration was serially two-fold diluted in Eppendorf tubes (2 mL volume) starting from 500 to 0.030 µg/mL for the pure compounds and from 500 to 0.007 µg/mL for the antibiotics.

After the completed test, performed as described above, the minimum inhibitory concentration was estimated from the spectrophotometric data as the smallest concentration that led to 90% or more growth inhibition. The absorbance of the broth was automatically subtracted from all test samples using the “zero” application in the Multiskan Sky (Thermo Fischer Scientific) spectrophotometer. The percentage growth was calculated according to the formula:

Percentage growth = (OD620 T − OD620 SC)/OD620 GC × 100 (2)

where OD620 T is the mean optical density at 620 nm of the duplicate test wells containing plant extract and bacteria, OD620 SC is the mean optical density of the duplicate wells containing the corresponding plant extract with broth but no bacteria, and OD620 GC is the mean optical density of the growth control. Therefore, the percentage growth inhibition was calculated as follows:

100 (percentage growth as defined for the growth control) − (OD620 T − OD620 SC)/OD620 GC × 100(3)

#### 4.3.5. Time-Kill Assay

The time-kill effects of the *S. aurita* methanol twig extract and a reference antibiotic, tetracycline hydrochloride, were measured against *B. cereus* and *S. aureus* using the microplate-based optical density (OD620) method, as described in Paragraph 4.3.4 for the turbidimetric microplate assay. For the time kill assay, 1 × MIC, 0.5 × MIC, and 0.25 × MIC concentrations of the extract and antibiotics were prepared. The tests were performed in flat-bottomed 96-well plates (Nunc, Nunclon). The dilutions of the twig extracts and antibiotics were prepared with sterile Mueller–Hinton broth. First, the bacteria growing on nutrient agar slants were taken with an inoculation loop and transferred into 20 mL of nutrient broth in an Erlenmeyer flask and grown overnight for 24 h at +37 °C with shaking at 200 rpm using an orbital incubator (Stuart^®^ SI500289, London, UK). The absorbance of 1 mL taken from the overnight bacterial culture was measured for turbidity at 625 nm, using a UV–visible spectrophotometer and the absorbance was adjusted to 0.1 at 625 nm (containing approximately 1.0 × 10^8^ CFU/mL) as performed in the microplate method described above. A total of 100 µL of the suspension at A_625_ = 0.1 was further diluted with 9.9 mL broth to obtain a 100-fold dilution which contains approximately 1 × 10^6^ CFU/mL. This dilution was used as the inoculum for the test. Then 100 µL of the bacterial suspension and 100 µL of Mueller–Hinton broth was pipetted in the growth control (GC) wells of the microplate. The *S. aurita* methanol twig extract was tested at its MIC, 0.5 × MIC, and 0.25 × MIC concentrations, which equaled to concentrations of 2500, 1250, and 625 µg/mL when tested against *B. cereus* and 1250, 625, and 313 µg/mL when tested against *S. aureus*. For the test (T) wells, we added 100 µL of the bacterial suspension and 100 µL of the following dilutions of the *S. aurita* extract or tetracycline hydrochloride (MIC, 0.5 × MIC, or 0.25 × MIC) in the wells according to the pipetting scheme for the specific plate. As for the microplate method described earlier, sample controls were used to subtract any absorbance at 620 nm resulting from the extract itself. After finishing the pipetting of the twig extracts and the antibiotic in the 96-well microplate, the OD620 at time zero (T0) was measured. At the starting point of the experiment, the OD 620 measurement value at T0 was 0.05. Following this, the microplate was incubated for 24 h at 37 °C, 350 RPM in a microplate shaker, and the OD620 was measured spectrophotometrically every second hour from 2 to 8 h and after 24 h. The results were presented as the change in OD620 over a time period of 24 h, and the growth inhibition of the plant extract was compared to that of tetracycline hydrochloride and to the growth control.

### 4.4. Methods of Analytical Chemistry

#### UHPLC-PDA-QTOF/MS

UPLC-PDA-QTOF/MS (ESI-mode) was used for the chemical profiling, identification, and molecular mass determination of compounds present in an antibacterial methanol twig extract of *S. aurita*. Some commercial reference standard compounds present in *Salix* species were also analyzed. For the UPLC-PDA-QTOF/MS (ESI- mode) analysis, 5 mg of the twig extracts were dissolved in 1 mL of methanol and milli-Q water at a ratio of 1:1. For the pure compounds, a 1 mg/mL concentration was prepared in methanol and milli-Q water at a ratio of 1:1. The samples were analyzed with UPLC-PDA-Synapt G2 QTOF/HDMS (Framingham, MA, USA), and a Waters reverse phase C18 column (50 × 2.1 mm, ø 1.7 μm) at 40 °C connected to an Acquity UPLC instrument (Waters, Framingham, MA, USA). A total of 2 μL of a 5 mg/mL concentration of the twig extracts in MeOH: H_2_O (1:1) was injected into the column and the flow rate was 0.6 mL/min. A gradient run from 5% B to 95% B in 9 min, switched back to 5% B and left to stabilize for 1 min was performed using a binary solvent system of A (0.1% formic acid in both milli-Q water (A)), and acetonitrile (B), with a total run time of 10 min. The UVλ absorption maxima spectra of the major compounds in the twig extracts were recorded using MassLynx software (V4.2, Waters, Framingham, MA, USA), and the compounds in the twig extracts were identified by comparing their retention times, their exact mass to charge ratios (*m*/*z*), and their UV spectra with those of commercial reference standards and also with previous data from the literature [26,38].

### 4.5. Statistical Analysis

All the data obtained from the antibacterial screening and extraction yields are expressed as mean ± standard error of mean (SEM). The diameters of the inhibition zones (IZDs) were obtained as means of 3 replicates (n = 3) and each of the experiments were performed twice independently. The percentage inhibition was obtained from the data of the duplicate experiment of the percentage growth (n = 2) using a coded Microsoft Excel and each of the experiments were performed twice independently. The percentage growth inhibition was presented as the mean of duplicates ± SEM.

## 5. Conclusions

In this study, polar twig extracts of *S. aurita*, *S. caprea*, and *S. pyrolifolia* inhibited the growth of *S. aureus*, *B. cereus*, *P. aeruginosa*, and *E. coli*. *S. aurita* and *S. pyrolifolia*, which have not been investigated before for their antimicrobial effects, revealed significant growth inhibitory activities against *Pseudomonas aeruginosa*. The phytochemical and structural investigation of the methanol extract of *S. aurita* revealed that it contained mostly proanthocyanidins (procyanidin B1 and procyanidin C1 and other procyanidin derivatives), flavonoids, and phenolic glucosides. From these results, it could be ascertained that *S. aurita*, *S. caprea*, and *S. pyrolifolia* may contain a high variety and concentration of polyphenolic compounds that may be explored as antibacterial agents or adjuvants, alone and in combination with conventional antibiotics, to combat multidrug-resistant bacteria. The significant antibacterial activity observed in the studied *Salix* extracts and the presence of procyanidins, flavonoids, and phenolic glycosides in the UPLC/QTOF-MS phytochemical analysis shows that *Salix* extracts could be utilized in the development of new antibacterial agents or antibiotic adjuvants for drug discovery. However, since the antibacterial effects of polyphenolic compounds such as procyanidins, flavonoids, and phenolic glucosides could be affected by the cell structure of the bacterium, the treatment time, and the level of exposure of the polyphenol on the bacterium, further analysis of the mechanism of action of these polyphenolic compounds as antibacterial agents is needed. The antibacterial mechanisms of the polyphenolic compounds on *S. aureus*, *B. cereus*, *P. aeruginosa*, and *E. coli* should be further studied in the aspects of the effects on the bacterial morphological structure, the cell wall, cell membrane, antibiofilm effects, quorum-sensing, and efflux pump activity. Furthermore, in vivo and in vitro antibacterial models and toxicity experiments should be conducted to ascertain the toxicity of these *Salix* extracts and to further evaluate if there are health implications of the extracts and their identified polyphenolic compounds. The phenolic polymers, procyanidin B1 and C1, identified in *S. aurita* for the first time, should be further analyzed quantitively to know the exact concentrations of individual procyanidins in *S. aurita* twigs. In depth knowledge about these identified polyphenolic compounds could be a lead to creating alternative novel therapeutic approaches and antibacterial agents to combat microbial resistance. In this context, it can be mentioned that the *Salix* species presented in this study can be sustainably grown on abandoned peatlands and could thus be used as fast-growing and low-demanding sustainable sources for antibacterial extracts and compounds. Importantly, the studied willow species can be sources of less toxic and more environmentally friendly antimicrobial agents.

## Figures and Tables

**Table 1:** Antibacterial effects of twig extracts of *S. aurita*, *S. caprea*, and *S. pyrolifolia*. The results were obtained with an agar diffusion and the turbidimetric microplate method. Significant results are marked with bold font.

Extracts/Pure Compounds	*P. aeruginosa* ATCC 27853			*S. aureus* ATCC 25923			*E. coli* ATCC P25922			*B. cereus* ATCC 10987		
	IZD	% GI	MIC	IZD	% GI	MIC	IZD	% GI	MIC	IZD	% GI	MIC
*S. aurita* H_2_O*	18.3 ± 0.3	**93.12**	2500	25.0 ± 0.0	78.89	>2500	18.3 ± 0.3	NT	NT	17.00 ± 0.0	82.09	>2500
*S. aurita* HH_2_O	20.8 ± 0.7	**97.19**	2500	20.83 ± 0.4	**93.35**	2500	NA	NA	NA	19.7 ± 0.5	**99.39**	2500
*S. aurita* Me*	24.3 ± 1.2	81.00	>2500	23.08 ± 0.5	**92.76**	1250	NA	NA	NA	21.67 ± 0.3	**118.19**	2500
*S. pyrolifolia* Me*	22.0 ± 0.0	**91.67**	2500	20.00 ± 00	**109.44**	2500	NA	NA	NA	22.00 ± 0.0	67.16	>2500
*S. pyrolifolia* HH_2_O	22.0 ± 0.0	85.39	>2500	**27.5 ± 0.5**	61.04	>2500	NA	NA	NA	20.00 ±0.0	83.09	>2500
*S. pyrolifolia* H_2_O*	18.00 ± 0.0	**102.86**	2500	**30.0 ± 0.0**	**91.98**	2500	NA	NA	NA	26.3 ± 0.3	82.59	>2500
*S. caprea* H_2_O*	22.00 ± 0.0	**96.48**	2500	NA	10.64	NA	22.00 ± 0.0	16.17	NA	18.00 ± 0.0	60.34	>2500
*S. caprea* Me*	20.0 ± 0.0	55.26	>2500	29.8 ± 0.3	**98.58**	2500	NA	NA	NA	**30.0 ± 0.0**	**95.93**	2500
*S. caprea* HH_2_O	20.3 ± 0.3	59.88	>2500	22.5 ± 0.5	53.79	>2500	NA	NA	NA	18.8 ± 0.3	66.53	>2500
Rifampicin	42.50± 0.4	101.08	31.25	42.50 ± 0.4	99.06	0.01	46.17 ± 2.3	99.75	15.63	NT	NT	0.244
Tetracycline	37.7 ± 0.9	99.97	62.5	52 ± 0.6	94.18	0.25	48.50 ± 1.2	97.44	0.98	44.67 ± 0.7	99.87	0.49
Naringenin	NT	39.1	>500	NT	NT	NT	NT	NT	NT	NT	NT	NT
Trans-p-HCA	25.67 ± 1.9	26.5	>500	25.67 ± 1.9	NT	NT	NT	NT	NT	18.83 ± 0.9	**99.13**	250
D (−)-Salicin	19.00 ± 0.0	26.12	>500	19.00 ± 0.0	NT	NT	NA	NT	NT	13.00 ± 0.0	NT	NT
Procyanidin B1	NT	48.8	>500	NT	NT	NT	NT	NT	NT	NT	**94.89**	250
Salicortin	NT	20.5	> 500	NT	NT	NT	NT	NT	NT	NT	68.19	>500
Taxifolin	NT	33.4	> 500	NT	NT	NT	NT	NT	NT	NT	**99.9**	250
Trans-m-HCA	NT	25.5	> 500	NT	NT	NT	NT	NT	NT	NT	70.62	>500
Acetyl-salicortin	NT	19.9	> 500	NT	NT	NT	NT	NT	NT	NT	75.9	>500
Catechin	NT	33.3	>500	NT	NT	NT	NT	NT	NT	NT	**98.3**	250

IZD: diameter of inhibition zone; % GI, percentage growth inhibition; MIC, minimum inhibitory concentration in µg/mL; H_2_O*, cold water maceration; HH_2_O, hot water decoction; Me*, cold methanol extract; NT, not tested; NA, not active. For the agar diffusion method, 200 µL of extracts of 50 mg/mL and compounds of 10 mg/mL were tested. For the microplate method, the extracts were first tested at 2500 µg/mL and twofold dilutions of active extracts were tested further to reach the MIC. The pure compounds were tested at 500 µg/mL initially and active extracts were twofold diluted to reach the MIC. The antibiotics were tested starting from 125 µg/mL and twofold dilutions were made to reach the MIC. HCA, hydroxycinnamic acid. The agar diffusion results are means ± SEM of triplicates. The percentage growth inhibition is reported as the mean ± SEM of duplicates. The experiments were repeated 2–3 times.

**Table 2:** UPLC/QTOF-MS data of polyphenolic compounds in a *S. aurita* methanol twig extract.

Name of Compound	R. Time	Measured Mass *m*/*z*	Chemical Formula	Calculated Mass *m*/*z*	Error (mDa)	Error (ppm)
Caffeoylhexose (**1**)	0.23	341.1083	C_15_H_18_O_9_	341.1089	0.2	0.6
Galloylglucose (**2**)	0.44	331.0660	C_13_H_16_O_10_	331.0665	−0.5	−1.5
Catechin–gallocatechin dimer (**3**)	0.50	593.1290	C_30_H_26_O_13_	593.1295	−0.5	−0.8
Protocatechuoylglucose (**4**)	0.54	315.0714	C_13_H_16_O_9_	315.0716	−0.2	−0.6
Epigallocatechin (**5**)	0.56	305.0661	C_15_H_14_O_7_	305.0661	0.0	0.0
Catechin–gallocatechin dimer (**6**)	0.62	593.1294	C_30_H_26_O_13_	593.1295	−0.1	−0.2
Catechin derivative (**7**)	0.64	289.0709	C_15_H_14_O_6_	289.0712	−0.3	−1.0
Salicin-7-sulfate (**8**)	0.68	365.0538	C_13_H_18_O_10_S	365.0542	−0.4	−1.1
Epigallocatechin-epicatechin-epicatechin (**9**)	0.75	881.1914	C_45_H_38_O_19_	881.1929	−1.5	−1.7
Salicin (**10**)	0.76	285.0977	C_13_H_18_O_7_	285.0977	0.0	0.0
Tryptophan (**11**)	0.78	203.0814	C_11_H_12_N_2_O_2_	203.0821	−0.7	−3.4
Caffeoylhexose isomer (**12**)	0.79	341.0872	C_15_H_18_O_9_	341.0873	−0.1	−0.3
Catechin–gallocatechin dimer (**13**)	0.85	593.1294	C_30_H_26_O_13_	593.1295	−0.1	−0.2
Procyanidin B1 (**14**)	0.88	577.1342	C_30_H_26_O_12_	577.1346	−0.2	−0.3
Salicylic acid glucoside (**15**)	0.92	299.0752	C_13_H_16_O_8_	299.0767	−1.5	−5.0
Catechin (**16**)	0.95	289.0705	C_15_H_14_O_6_	289.0712	−0.7	−2.4
Coumaric acid 2-glucoside (**17**)	1.01	325.0922	C_15_H_18_O_8_	325.0923	−0.1	−0.3
Flavonoid pentose (**18**)	1.06	491.1765	C_21_H_32_O_13_	491.1765	0.0	0.0
Procyanidin C1 (**19**)	1.14	865.1968	C_45_H_38_O_18_	865.1980	−1.2	−1.4
Benzyl-ß-primeveroside (**20**)	1.21	401.1452	C_18_H_26_O_10_	401.1448	0.4	1.0
Unknown (**21**)	1.31	357.0813	C_15_H_18_O_10_	357.0822	−0.9	−2.5
Procyanidin B1 dimer (**22**)	1.39	577.1322	C_30_H_26_O_12_	577.1346	−2.4	−4.2
Unknown (**23**)	1.57	715.1660	C_37_H_32_O_15_	715.1663	−0.3	−0.4
Rutin syn. Quercetin rutinoside (**24**)	1.65	609.1445	C_27_H_30_O_16_	609.1456	−1.1	−1.8
Quercetin-3-O-glucoside (**25**)	1.68	463.0872	C_21_H_20_O_12_	463.0877	−0.5	−1.1
Taxifolin (**26**)	1.69	303.0500	C_15_H_12_O_7_	303.0505	−0.5	−1.6
Catechin-rutinoside (**27**)	1.72	593.1500	C_27_H_30_O_15_	593.1506	−0.6	−1.0
Salicortin (**28**)	1.79	423.1288	C_20_H_24_O_10_	423.1291	−0.3	−0.7
Naringenin-7-glucoside (**29**)	1.84	433.0762	C_20_H_18_O_11_	433.0771	−0.9	−2.1
Taxifolin-xylopyranoside (**30**)	1.89	435.0917	C_20_H_20_O_11_	435.0927	−1.0	−2.3
Naringenin (**31**)	1.94	271.0599	C_15_H_12_O_5_	271.0606	−0.7	−2.6
Isorhamnetin glycoside (**32**)	2.00	477.1024	C_22_H_22_O_12_	477.1033	−0.9	−1.9
Chrysoeriol-7-glucoside (**33**)	2.22	461.1084	C_22_H_22_O_11_	461.1084	0.0	0.0
2′-O-Acetylsalicortin (**34a**)	2.32	465.1394	C_22_H_26_O_11_	465.1397	−0.3	−0.6
Salireposide (**34b**)	2.32	405.1174	C_20_H_22_O_9_	405.1186	−1.2	−3.0
Salicortin derivative (**35a**)	2.40	423.1646	C_21_H_28_O_9_	423.1655	−0.9	−2.1
Unknown (**35b**)	2.40	403.1603	C_18_H_28_O_10_	403.1604	−0.1	−0.2
Luteolin (**36**)	2.54	285.0391	C_15_H_10_O_6_	285.0399	−0.8	−2.8
Unknown (**37**)	2.67	431.1349	C_11_H_15_NO_3_	431.1350	−1.0	−2.1
Acetyl-salicortin derivative (**38**)	3.03	465.1387	C_22_H_26_O_11_	465.1397	−1.0	−2.1
Salicortin derivative (**39**)	3.07	423.1289	C_20_H_24_O_10_	423.1291	−0.2	−0.5
Procyanidin B1 dimer (**40**)	3.12	577.1363	C_30_H_26_O_12_	577.1346	1.7	2.9
Tremulacin (**41**)	3.24	527.1558	C_27_H_28_O_11_	527.1553	0.5	0.9
Salicortin derivative (**42**)	3.36	423.1291	C_20_H_24_O_10_	423.1291	0.0	0.0
Unknown (**43**)	3.46	329.2322	C_18_H_34_O_5_	329.2328	−0.6	−1.8
Methylated catechin glucopyranoside (**44**)	3.65	465.1389	C_22_H_26_O_11_	465.1397	−0.8	−1.7

UPLC/QTOF-MS in negative ion mode was used. The mass accuracy is explained as parts per million, ppm. All the identified compounds are numbered in the table as in the text.

## Data Availability

All the data presented here are also available upon request.
